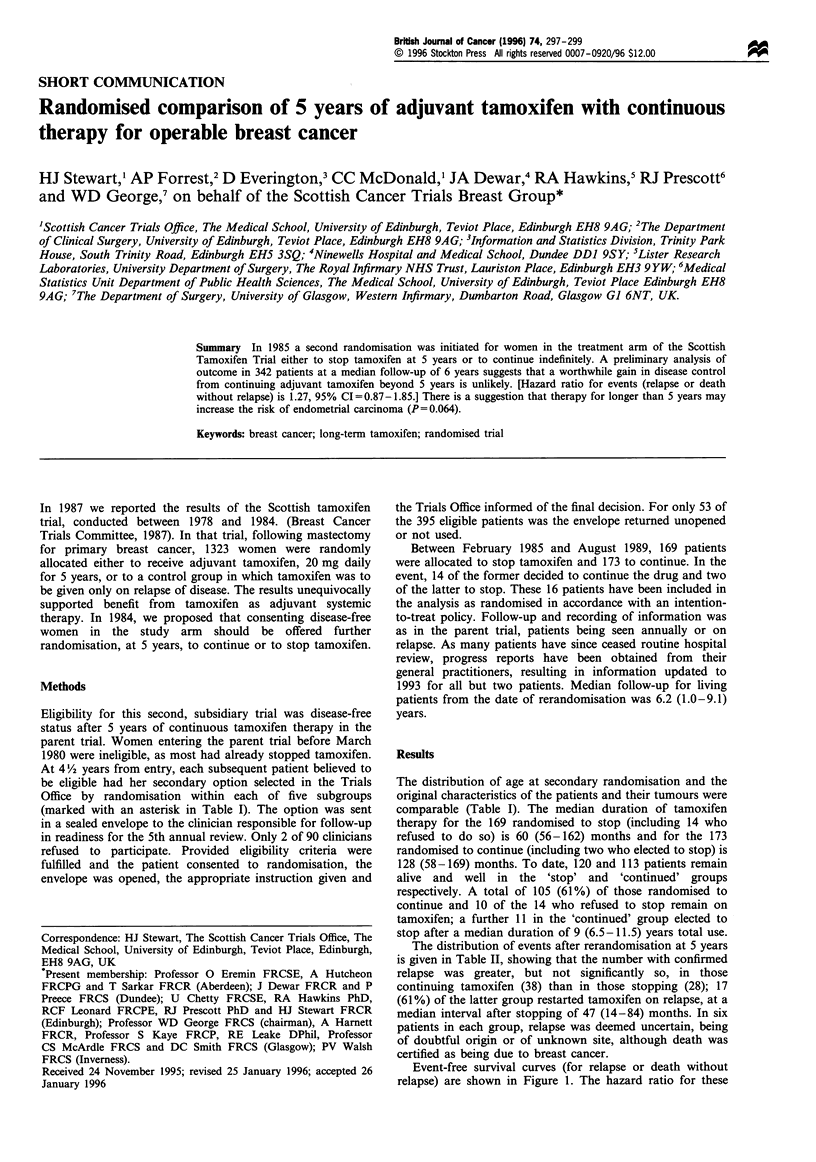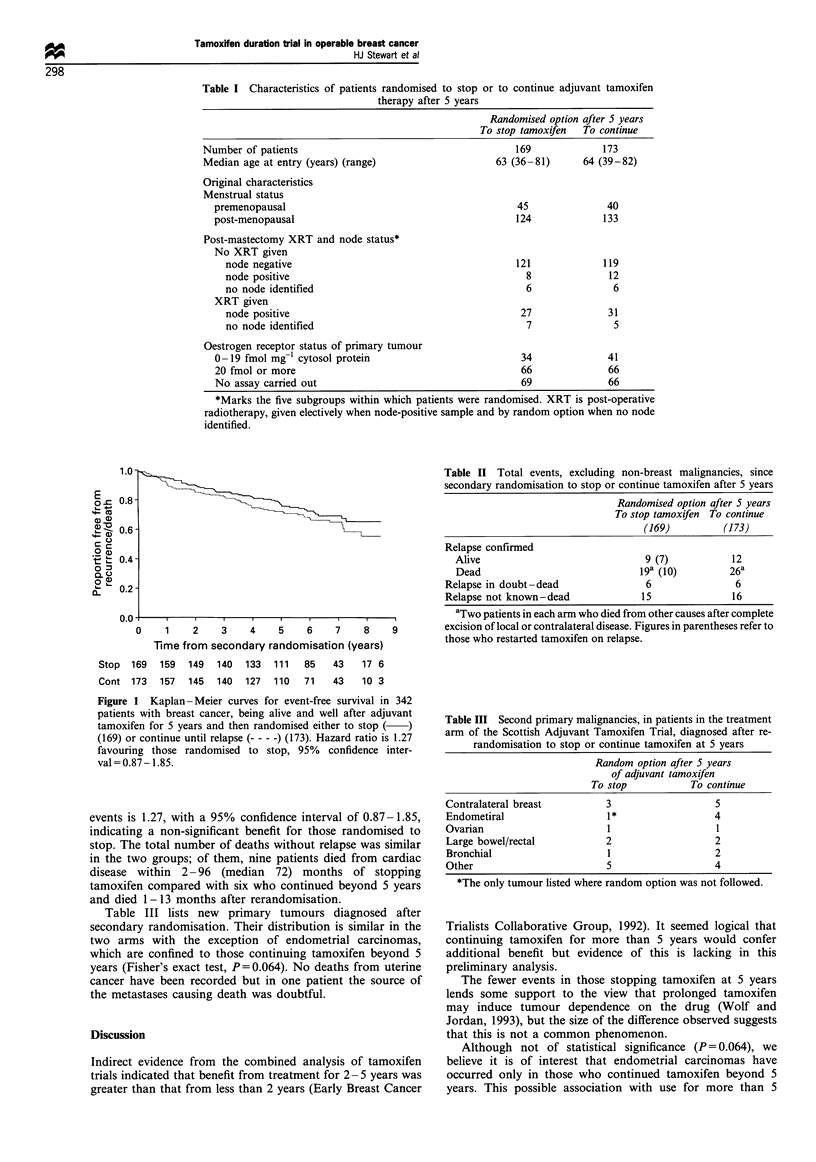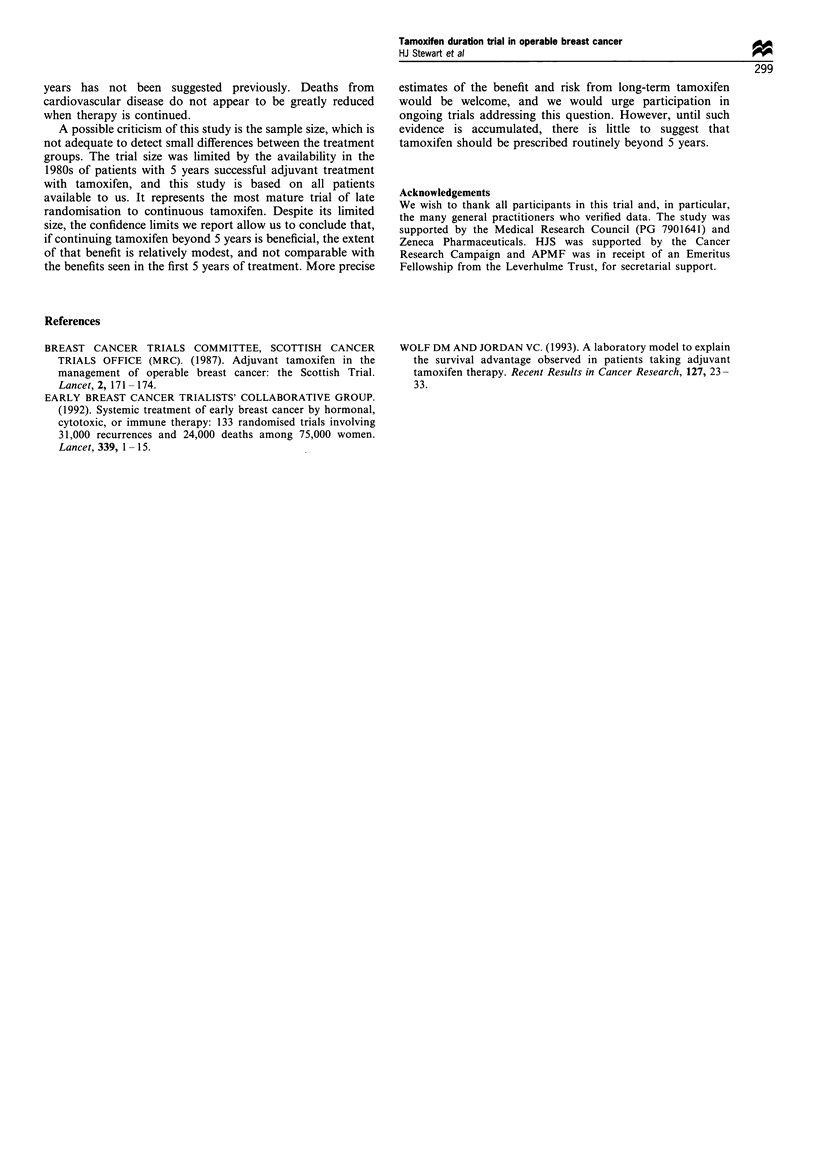# Randomised comparison of 5 years of adjuvant tamoxifen with continuous therapy for operable breast cancer. The Scottish Cancer Trials Breast Group.

**DOI:** 10.1038/bjc.1996.356

**Published:** 1996-07

**Authors:** H. J. Stewart, A. P. Forrest, D. Everington, C. C. McDonald, J. A. Dewar, R. A. Hawkins, R. J. Prescott, W. D. George

**Affiliations:** Scottish Cancer Trials Office, Medical School, University of Edinburgh, UK.

## Abstract

In 1985 a second randomisation was initiated for women in the treatment arm of the Scottish Tamoxifen Trial either to stop tamoxifen at 5 years or to continue indefinitely. A preliminary analysis of outcome in 342 patients at a median follow-up of 6 years suggests that a worthwhile gain in disease control from continuing adjuvant tamoxifen beyond 5 years is unlikely. [Hazard ratio for events (relapse or death without relapse) is 1.27, 95% CI = 0.87 - 1.85.] There is a suggestion that therapy for longer than 5 years may increase the risk of endometrial carcinoma (P = 0.064).


British Journal of Cancer (1996) 74, 297-299
© 1996 Stockton Press All rights reserved 0007-0920/96 $12.00

SHORT COMMUNICATION

### Randomised comparison of 5 years of adjuvant tamoxifen with continuous therapy for operable breast cancer

HJ Stewart,[1] AP Forrest,[2] D Everington,[3] CC McDonald,[1] JA Dewar,[4] RA Hawkins,[5] RJ Prescott[6] and WD George,[7] on behalf of the Scottish Cancer Trials Breast Group*

[1]*Scottish Cancer Trials Office, The Medical School, University of Edinburgh, Teviot Place, Edinburgh EH8 9AG;* [2]*The Department of Clinical Surgery, University of Edinburgh, Teviot Place, Edinburgh EH8 9AG;* [3]*Information and Statistics Division, Trinity Park House, South Trinity Road, Edinburgh EH5 3SQ;* [4]*Ninewells Hospital and Medical School, Dundee DD1 9SY;* [5]*Lister Research Laboratories, University Department of Surgery, The Royal Infirmary NHS Trust, Lauriston Place, Edinburgh EH3 9YW;* [6]*Medical Statistics Unit Department of Public Health Sciences, The Medical School, University of Edinburgh, Teviot Place Edinburgh EH8 9AG;* [7]*The Department of Surgery, University of Glasgow, Western Infirmary, Dumbarton Road, Glasgow G1 6NT, UK.*

**Summary** In 1985 a second randomisation was initiated for women in the treatment arm of the Scottish Tamoxifen Trial either to stop tamoxifen at 5 years or to continue indefinitely. A preliminary analysis of outcome in 342 patients at a median follow-up of 6 years suggests that a worthwhile gain in disease control from continuing adjuvant tamoxifen beyond 5 years is unlikely. [Hazard ratio for events (relapse or death without relapse) is 1.27, 95% CI=0.87-1.85.] There is a suggestion that therapy for longer than 5 years may increase the risk of endometrial carcinoma ($P$=0.064).

**Keywords:** breast cancer; long-term tamoxifen; randomised trial

In 1987 we reported the results of the Scottish tamoxifen trial, conducted between 1978 and 1984. (Breast Cancer Trials Committee, 1987). In that trial, following mastectomy for primary breast cancer, 1323 women were randomly allocated either to receive adjuvant tamoxifen, 20 mg daily for 5 years, or to a control group in which tamoxifen was to be given only on relapse of disease. The results unequivocally supported benefit from tamoxifen as adjuvant systemic therapy. In 1984, we proposed that consenting disease-free women in the study arm should be offered further randomisation, at 5 years, to continue or to stop tamoxifen.

### Methods

Eligibility for this second, subsidiary trial was disease-free status after 5 years of continuous tamoxifen therapy in the parent trial. Women entering the parent trial before March 1980 were ineligible, as most had already stopped tamoxifen. At 4½ years from entry, each subsequent patient believed to be eligible had her secondary option selected in the Trials Office by randomisation within each of five subgroups (marked with an asterisk in Table I). The option was sent in a sealed envelope to the clinician responsible for follow-up in readiness for the 5th annual review. Only 2 of 90 clinicians refused to participate. Provided eligibility criteria were fulfilled and the patient consented to randomisation, the envelope was opened, the appropriate instruction given and the Trials Office informed of the final decision. For only 53 of the 395 eligible patients was the envelope returned unopened or not used.

Between February 1985 and August 1989, 169 patients were allocated to stop tamoxifen and 173 to continue. In the event, 14 of the former decided to continue the drug and two of the latter to stop. These 16 patients have been included in the analysis as randomised in accordance with an intention-to-treat policy. Follow-up and recording of information was as in the parent trial, patients being seen annually or on relapse. As many patients have since ceased routine hospital review, progress reports have been obtained from their general practitioners, resulting in information updated to 1993 for all but two patients. Median follow-up for living patients from the date of rerandomisation was 6.2 (1.0-9.1) years.

### Results

The distribution of age at secondary randomisation and the original characteristics of the patients and their tumours were comparable (Table I). The median duration of tamoxifen therapy for the 169 randomised to stop (including 14 who refused to do so) is 60 (56-162) months and for the 173 randomised to continue (including two who elected to stop) is 128 (58-169) months. To date, 120 and 113 patients remain alive and well in the 'stop' and 'continued' groups respectively. A total of 105 (61%) of those randomised to continue and 10 of the 14 who refused to stop remain on tamoxifen; a further 11 in the 'continued' group elected to stop after a median duration of 9 (6.5-11.5) years total use.

The distribution of events after rerandomisation at 5 years is given in Table II, showing that the number with confirmed relapse was greater, but not significantly so, in those continuing tamoxifen (38) than in those stopping (28); 17 (61%) of the latter group restarted tamoxifen on relapse, at a median interval after stopping of 47 (14-84) months. In six patients in each group, relapse was deemed uncertain, being of doubtful origin or of unknown site, although death was certified as being due to breast cancer.

Event-free survival curves (for relapse or death without relapse) are shown in Figure 1. The hazard ratio for these

Correspondence: HJ Stewart, The Scottish Cancer Trials Office, The Medical School, University of Edinburgh, Teviot Place, Edinburgh, EH8 9AG, UK

*Present membership: Professor O Eremin FRCSE, A Hutcheon FRCPG and T Sarkar FRCR (Aberdeen); J Dewar FRCR and P Preece FRCS (Dundee); U Chetty FRCSE, RA Hawkins PhD, RCF Leonard FRCPE, RJ Prescott PhD and HJ Stewart FRCR (Edinburgh); Professor WD George FRCS (chairman), A Harnett FRCR, Professor S Kaye FRCP, RE Leake DPhil, Professor CS McArdle FRCS and DC Smith FRCS (Glasgow); PV Walsh FRCS (Inverness).

Received 24 November 1995; revised 25 January 1996; accepted 26 January 1996

**Table I** Characteristics of patients randomised to stop or to continue adjuvant tamoxifen therapy after 5 years

| | *Randomised option after 5 years* | |
|---|---|---|
| | *To stop tamoxifen* | *To continue* |
| Number of patients | 169 | 173 |
| Median age at entry (years) (range) | 63 (36–81) | 64 (39–82) |
| Original characteristics | | |
| Menstrual status | | |
| premenopausal | 45 | 40 |
| post-menopausal | 124 | 133 |
| Post-mastectomy XRT and node status* | | |
| No XRT given | | |
| node negative | 121 | 119 |
| node positive | 8 | 12 |
| no node identified | 6 | 6 |
| XRT given | | |
| node positive | 27 | 31 |
| no node identified | 7 | 5 |
| Oestrogen receptor status of primary tumour | | |
| 0–19 fmol $mg^{-1}$ cytosol protein | 34 | 41 |
| 20 fmol or more | 66 | 66 |
| No assay carried out | 69 | 66 |

*Marks the five subgroups within which patients were randomised. XRT is post-operative radiotherapy, given electively when node-positive sample and by random option when no node identified.

**Figure 1** Kaplan–Meier curves for event-free survival in 342 patients with breast cancer, being alive and well after adjuvant tamoxifen for 5 years and then randomised either to stop (——) (169) or continue until relapse (- - - -) (173). Hazard ratio is 1.27 favouring those randomised to stop, 95% confidence interval = 0.87–1.85.

events is 1.27, with a 95% confidence interval of 0.87–1.85, indicating a non-significant benefit for those randomised to stop. The total number of deaths without relapse was similar in the two groups; of them, nine patients died from cardiac disease within 2–96 (median 72) months of stopping tamoxifen compared with six who continued beyond 5 years and died 1–13 months after rerandomisation.

Table III lists new primary tumours diagnosed after secondary randomisation. Their distribution is similar in the two arms with the exception of endometrial carcinomas, which are confined to those continuing tamoxifen beyond 5 years (Fisher's exact test, $P = 0.064$). No deaths from uterine cancer have been recorded but in one patient the source of the metastases causing death was doubtful.

### Discussion

Indirect evidence from the combined analysis of tamoxifen trials indicated that benefit from treatment for 2–5 years was greater than that from less than 2 years (Early Breast Cancer Trialists Collaborative Group, 1992). It seemed logical that continuing tamoxifen for more than 5 years would confer additional benefit but evidence of this is lacking in this preliminary analysis.

The fewer events in those stopping tamoxifen at 5 years lends some support to the view that prolonged tamoxifen may induce tumour dependence on the drug (Wolf and Jordan, 1993), but the size of the difference observed suggests that this is not a common phenomenon.

Although not of statistical significance ($P = 0.064$), we believe it is of interest that endometrial carcinomas have occurred only in those who continued tamoxifen beyond 5 years. This possible association with use for more than 5

**Table II** Total events, excluding non-breast malignancies, since secondary randomisation to stop or continue tamoxifen after 5 years

| | *Randomised option after 5 years* | |
|---|---|---|
| | *To stop tamoxifen (169)* | *To continue (173)* |
| Relapse confirmed | | |
| Alive | 9 (7) | 12 |
| Dead | 19[a] (10) | 26[a] |
| Relapse in doubt–dead | 6 | 6 |
| Relapse not known–dead | 15 | 16 |

[a]Two patients in each arm who died from other causes after complete excision of local or contralateral disease. Figures in parentheses refer to those who restarted tamoxifen on relapse.

**Table III** Second primary malignancies, in patients in the treatment arm of the Scottish Adjuvant Tamoxifen Trial, diagnosed after re-randomisation to stop or continue tamoxifen at 5 years

| | *Random option after 5 years of adjuvant tamoxifen* | |
|---|---|---|
| | *To stop* | *To continue* |
| Contralateral breast | 3 | 5 |
| Endometiral | 1* | 4 |
| Ovarian | 1 | 1 |
| Large bowel/rectal | 2 | 2 |
| Bronchial | 1 | 2 |
| Other | 5 | 4 |

*The only tumour listed where random option was not followed.

years has not been suggested previously. Deaths from cardiovascular disease do not appear to be greatly reduced when therapy is continued.

A possible criticism of this study is the sample size, which is not adequate to detect small differences between the treatment groups. The trial size was limited by the availability in the 1980s of patients with 5 years successful adjuvant treatment with tamoxifen, and this study is based on all patients available to us. It represents the most mature trial of late randomisation to continuous tamoxifen. Despite its limited size, the confidence limits we report allow us to conclude that, if continuing tamoxifen beyond 5 years is beneficial, the extent of that benefit is relatively modest, and not comparable with the benefits seen in the first 5 years of treatment. More precise estimates of the benefit and risk from long-term tamoxifen would be welcome, and we would urge participation in ongoing trials addressing this question. However, until such evidence is accumulated, there is little to suggest that tamoxifen should be prescribed routinely beyond 5 years.

**Acknowledgements**
We wish to thank all participants in this trial and, in particular, the many general practitioners who verified data. The study was supported by the Medical Research Council (PG 7901641) and Zeneca Pharmaceuticals. HJS was supported by the Cancer Research Campaign and APMF was in receipt of an Emeritus Fellowship from the Leverhulme Trust, for secretarial support.

### References

BREAST CANCER TRIALS COMMITTEE, SCOTTISH CANCER TRIALS OFFICE (MRC). (1987). Adjuvant tamoxifen in the management of operable breast cancer: the Scottish Trial. *Lancet*, **2**, 171–174.

EARLY BREAST CANCER TRIALISTS' COLLABORATIVE GROUP. (1992). Systemic treatment of early breast cancer by hormonal, cytotoxic, or immune therapy: 133 randomised trials involving 31,000 recurrences and 24,000 deaths among 75,000 women. *Lancet*, **339**, 1–15.

WOLF DM AND JORDAN VC. (1993). A laboratory model to explain the survival advantage observed in patients taking adjuvant tamoxifen therapy. *Recent Results in Cancer Research*, **127**, 23–33.